# Treatment Selection for Patients with HER2-Negative Metastatic Gastric Cancer Expressing Claudin 18.2 and PD-L1

**DOI:** 10.3390/cancers17071120

**Published:** 2025-03-27

**Authors:** Yusuke Miyajima, Takeshi Kawakami

**Affiliations:** Division of Gastrointestinal Oncology, Shizuoka Cancer Center, Shizuoka 411-8777, Japan; y38.miyajima@gmail.com

**Keywords:** claudin18.2, combined positive score (CPS), gastric cancer, immune checkpoint inhibitor, zolbetuximab

## Abstract

Combination therapy of chemotherapy and zolbetuximab demonstrated a significant survival benefit compared to chemotherapy alone in patients with human epidermal growth factor receptor 2 (HER2)-negative, claudin (CLDN) 18.2–positive metastatic gastric cancer (mGC). Consequently, it has been approved as a standard first-line therapy for these patients. Combination therapy of chemotherapy and immune checkpoint inhibitors (ICIs)—either nivolumab or pembrolizumab—is a standard first-line therapy for patients with HER2-negative mGCs that are positive for programmed death-ligand 1 (PD-L1) expression, as defined by a combined positive score (CPS). Although approximately 13–22% of CLDN-positive mGCs are also CPS-positive, optimal treatment for mGC patients expressing both CLDN and PD-L1 remains undetermined due to the absence of direct comparative studies between zolbetuximab and ICIs. Treatment selection under this condition has become a critical issue. In this review, we discuss the appropriate treatment selection for HER2-negative mGC patients who are double-positive for CLDN 18.2 and PD-L1 based on clinical data and differences in the mechanism of action and safety profile between zolbetuximab and ICI.

## Simple Summary

Zolbetuximab, in combination with chemotherapy, has become a standard first-line treatment for patients with HER2-negative, claudin (CLDN) 18.2-positive metastatic gastric cancer (mGC). Chemotherapy plus immune checkpoint inhibitors (ICIs)—either nivolumab or pembrolizumab—is also a standard first-line treatment for HER2-negative mGC that is positive for programmed death-ligand 1 (PD-L1) combined positive score (CPS). Approximately 13–22% of CLDN-positive mGCs are also CPS-positive, suggesting that several patients with CLDN-positive mGC may receive optimal survival benefits from ICI plus chemotherapy. However, the selection of optimal treatment for this population has not been established due to the lack of direct comparative studies. In this review, we discuss appropriate treatment selection based on clinical data and differences in the mechanism of action and safety profile between zolbetuximab and ICI.

## 1. Introduction

The standard first-line treatment for gastric cancer with metastasis (mGC) is a combination therapy of fluoropyrimidine plus platinum agents with or without a molecular targeting agent according to biomarker status [1,2,3,4]. For patients with human epidermal growth factor receptor 2 (HER2)-positive mGC, trastuzumab plus chemotherapy has been the standard first-line therapy [1,2,3,4,5]. Recently, the KEYNOTE-811 trial demonstrated that the combination therapy of pembrolizumab and trastuzumab plus chemotherapy significantly improved overall survival (OS) and progression-free survival (PFS) in patients with programmed death-ligand 1 (PD-L1) expression (combined positive score: CPS ≥ 1) compared with trastuzumab plus chemotherapy. Currently, combination therapy of pembrolizumab and chemotherapy plus trastuzumab has emerged as a new standard treatment for HER2-positive mGCs with CPS ≥ 1 in the US and European countries [1,2,4,6].

The development of treatments for patients with HER2-negative mGC had stalled for some time. The CheckMate-649 trial demonstrated the clinical benefit of nivolumab plus chemotherapy combination therapy on OS and PFS, particularly in patients with CPS ≥ 5 [7]. Additionally, the ATTRACTION-4 trial, conducted in Asian countries, also demonstrated significant survival benefits in PFS [8]. Although the CPS positivity cutoff varies across countries, nivolumab plus chemotherapy has become the first-line standard of care for gastric cancer. Recently, pembrolizumab plus chemotherapy has emerged as another first-line standard treatment for HER2-negative mGC in the US, Europe, and Japan, based on results from the KEYNOTE-859 trial [2,3,9,10].

Zolbetuximab, targeting claudin (CLDN) 18.2, a tight junction molecule predominantly expressed in gastric epithelium [11,12], in combination with chemotherapy demonstrated significant improvements in PFS and OS for patients with HER2-negative, CLDN18.2-positive mGC in SPOTLIGHT and GLOW trials [13,14]. Zolbetuximab plus chemotherapy has emerged as the first-line standard of care for HER2-negative CLDN-positive mGC in Japan in 2024, followed by the US and Europe.

Although approximately 13–22% of CLDN-positive mGCs are also CPS-positive [13,14,15,16,17,18,19], optimal treatment selection for this population remains undetermined due to the absence of direct comparative studies between zolbetuximab and ICIs in combination with chemotherapy. Some patients may receive a greater survival benefit from ICI plus chemotherapy. For example, approximately 5–17% of CLDN-positive mGCs were microsatellite instability-high (MSI-H) or deficient mismatch repair (dMMR) [15,17,18,19], a strong predictive factor for ICI, suggesting these patients may benefit more from ICI plus chemotherapy. Furthermore, the hazard ratio (HR) for OS for ICI plus chemotherapy vs. chemotherapy alone in the CPS ≥ 10 subgroup in the CheckMate-649 trial tended to be lower than the HR for zolbetuximab plus chemotherapy vs. chemotherapy alone [20,21]. Since zolbetuximab plus chemotherapy and ICI plus chemotherapy demonstrate different efficacy profiles, optimal treatment may vary depending on individual circumstances and therapeutic goals. This review discusses optimal treatment selection for patients with CLDN 18.2-positive and PD-L1-positive metastatic gastric cancer.

## 2. Biomarkers in HER2-Negative Gastric Cancer

### 2.1. PD-L1 CPS

PD-L1 expression is assessed as CPS, which is defined as the ratio of the number of PD-L1-positive tumor cells, lymphocytes, and macrophages to the total number of tumor cells multiplied by 100 [22,23]. CPS is evaluated by immunohistochemistry (IHC) staining with PD-L1 antibody. The Dako PD-L1 IHC 28-8 pharmDx assay (Dako; Santa Clara, CA, USA) is used for nivolumab, with cutoff points of 1% and 5% [7]. The Dako PD-L1 IHC 22C3 pharmDx assay (Dako; Santa Clara, CA, USA) is used for pembrolizumab, and its cutoff points are 1% and 10% [9]. A high concordance was also observed between CPS assessed with the 28-8 and 22C3 antibodies [24,25,26]. Since CPS evaluated using these assays may yield comparable results, it seems reasonable to examine only one of them in clinical practice. However, it is important to take into account the fact that the cutoff values differ between these assays.

The interaction of programmed cell death-1 (PD-1) (expressed on the surface of cytotoxic T lymphocytes) with PD-L1 on antigen-presenting cells is one of the main mechanisms of immune modulation with immune checkpoints. Essentially, malignant cells with mutable neoantigens are recognized and removed by immune cells; however, malignant cells can upregulate PD-L1 expression following exposure to cytokines or other signaling, leading to stimulation of immune checkpoint inhibitory pathways [27]. Moreover, tumor cells promote an immunosuppressive tumor microenvironment (TME), in which PD-L1 expression is also upregulated in some immune cells, including dendritic cells, macrophages, and T cells [27,28,29], thus leading to immune evasion and tumor growth. Anti-PD-1 antibodies, including nivolumab and pembrolizumab, block these immunosuppressive pathways and enhance the host immune system, inducing antitumor effects through immune system-mediated cell death [30,31,32,33].

The prevalence of HER2-negative mGCs expressing PD-L1 has been reported as 16–49% with CPS ≥ 10, 60% with CPS ≥ 5, and 78–82% with CPS ≥ 1 [7,8,9]. The survival benefit of anti-PD-1 antibody plus chemotherapy tends to be greater with higher CPS [22,34,35]. As several studies have suggested intratumoral and spatial heterogeneity of CPS and discrepancy in CPS between pathologists—particularly when using a low cutoff value—the reliability of CPS has been questioned [24,36,37]. However, recent studies have suggested high CPS concordance among trained pathologists [24,38].

### 2.2. MSI-H/dMMR

Mismatch repair (MMR) genes, including *MLH1*, *MSH2*, *PMS2*, and *MSH6*, act in deoxyribonucleic acid (DNA) repair pathways in healthy cells [27,39]. Loss of MMR gene function, dMMR, is associated with MSI-H status [27,40]. Most sporadic MSI-H/dMMR gastric cancers are developed through the epigenetic silencing of the *MLH1* gene due to hypermethylation of the CpG island in its promoter region. On the other hand, germline mutations of the MMR genes are the main cause of Lynch syndrome [27,41,42,43]. MSI-H/dMMR tumors are related to increased production of neoantigens due to high tumor mutational burden, contributing to activated tumor-related immune response [27].

MSI status is evaluated by analyzing tumor DNA fragment length using polymerase chain reaction (PCR), while MMR status is assessed by IHC staining of MLH1, MSH2, MSH6, PMS1, and PMS2 proteins. The overall concordance between these tests was reported to be 97.5% [44]. The median turnaround time for MSI testing using the PCR method is 7 to 24 days, and MMR testing is considered shorter [41,45].

The prevalence of MSI-H/dMMR tumors ranges from 5% to 24% among any stage of gastric cancers and varies across countries: 5.0–18.7% in North America, 10.0–23.5% in European countries, and 5.6–13.8% in Asian countries [41,43,46]. These tumors are associated with female gender, older adults, lower stomach, intestinal-type histology, HER2-negative status, early-stage disease, and absence of liver metastasis [41,42,43,46]. If limited to stage IV disease, the incidence of MSI-H/dMMR ranges from 3% to 10% in mGCs [7,22,41,47]. Some patients with MSI-H/dMMR are CPS-negative [9,20,48]. MSI-H/dMMR is considered a strong predictive factor for ICI with or without chemotherapy and is, therefore, useful in drug selection [22,47,49,50].

### 2.3. Claudin 18.2

Claudin (CLDN) was first reported in 1998 as a component molecule of tight junction proteins by Tsukita et al. [51,52]. Currently, 26 human CLDN family molecules have been identified. In each epithelial tissue, various CLDN proteins are expressed simultaneously, regulating intercellular adhesion, tissue permeability, paracellular transport, and signal transduction. For example, CLDN 1, 5, and 6 contribute to glomerular filtration in the kidney. CLDN 1, 3, and 5 are involved in the regulation of vessel permeability in endothelial tissue [11,12,15,53,54]. CLDN 18 is one of them, and two splice variants of CLDN 18 were identified: CLDN 18.1 and CLDN 18.2. CLDN 18.1 is highly expressed in the lung alveolar epithelium but is not expressed in the gastric epithelium. CLDN 18.2 is predominantly expressed in normal gastric mucosal epithelial cells and is preserved even during malignant transformation [11,12,55,56]. In nonmalignant mucosa, CLDN 18.2 is located in the paracellular space, consisting of a tight junction complex. As carcinogenesis progresses, loss of cell polarity due to disruption of tight junction exposes CLDN 18.2 on the surface of malignant cells, which enables targeted antibodies to recognize tumor cells expressing CLDN 18.2 [12,51,57,58,59]. The prevalence of CLDN positivity is reported as 24–39% in mGC and 42.3% when limited to HER2-negative mGC [13,14,15,16,17,60]. CLDN expression is associated with age, sex, and tissue type: CLDN-positive tumors are more frequently observed in younger individuals, women, Borrmann type 4 tumors, and tumors with diffuse type or signet ring cell histology [16,61,62].

CLDN 18.2 expression in GC was assessed by IHC staining using 43-14A antibody and the VENTANA CLDN18 (43-14A) RxDx assay (Roche Diagnostic Solutions; Tucson, AZ, USA), which recognizes the intracellular domain shared by CLDN 18.1 and CLDN 18.2 [51]. The CLDN-positive criterion is defined as moderate to strong membrane staining, with an IHC score of 2+ or 3+, in ≥75% of tumor cells based on FAST trial results [13,14,51]. The spatial and intratumoral heterogeneity of CLDN expression in mGCs remains controversial [16,17,63,64].

## 3. ICI Plus Chemotherapy for HER2-Negative Gastric Cancer

Some cytotoxic agents, including platinum drugs, induce immunogenic cell death (ICD), which can activate the immune system against tumor antigens through the release of damage-associated molecular patterns (DAMPs) and stimulation of CD8^+^ dendritic cells [65,66,67]. It has also been suggested that ICD induced by cytotoxic chemotherapy may enhance the antitumor effect of ICIs, which improves the immune response to tumor cells [65,68]. Several clinical trials have been conducted to evaluate the survival benefit of combination chemotherapy with ICI for previously untreated HER2-negative mGCs [7,8,69,70].

In the CheckMate-649 trial, nivolumab plus chemotherapy (CAPOX [capecitabine and oxaliplatin] or FOLFOX [folinic acid, 5-fluorouracil, and oxaliplatin]) vs. chemotherapy alone was evaluated in previously untreated HER2-negative metastatic gastric or gastroesophageal junction (mG/GEJ) cancer. Nivolumab plus chemotherapy significantly prolonged median OS (14.4 vs. 11.1 months; HR 0.71; 98.4% CI 0.59–0.86) and median PFS (7.7 vs. 6.0 months; HR 0.68; 98% CI 0.56–0.81) in the CPS ≥ 5 group as dual primary endpoints. The overall response rate (ORR) of nivolumab plus chemotherapy tended to be higher in the CPS ≥ 5 group (60% vs. 45%) and in the overall randomized population (58% vs. 46%). An exploratory analysis indicated that differences in OS, PFS, and ORR were not significant in the CPS < 5 and CPS < 1 groups; however, ORR tended to be approximately 10% higher with nivolumab plus chemotherapy than without nivolumab, even in the CPS < 5 and CPS < 1 groups [7].

In the ATTRACTION-4 trial conducted in Asian countries, nivolumab plus chemotherapy (CAPOX or SOX [S-1 plus oxaliplatin]) was evaluated vs. chemotherapy alone in previously untreated HER2-negative G/GEJ cancer patients. Patients were eligible regardless of CPS, and analysis according to CPS was not performed in this trial. Nivolumab plus chemotherapy significantly improved median PFS (10.45 vs. 8.34 months; HR 0.68; 98.51% CI 0.51–0.90), whereas the difference in median OS was not significant (17.5 vs. 17.2 months; HR 0.68; 95% CI 0.75–1.08) in the overall randomized population. The ORR was significantly higher with nivolumab plus chemotherapy (57.5% vs. 47.8%; *p* = 0.0088) [8].

Nivolumab plus chemotherapy demonstrated significant survival benefits, particularly in patients with tumor CPS ≥ 5, while survival benefits in patients with tumor CPS < 5 remained unclear, resulting in varying regulatory approval status and recommendations across countries [71].

The efficacy of pembrolizumab plus chemotherapy was evaluated in the KEYNOTE-062 trial. Patients with previously untreated mG/GEJ cancers with CPS ≥ 1 were eligible and randomized to receive either pembrolizumab monotherapy, pembrolizumab plus chemotherapy (FP [5-fluorouracil and cisplatin] or XP [capecitabine and cisplatin]), or chemotherapy plus placebo. Pembrolizumab plus chemotherapy showed no significant improvement in OS (HR 0.85, 95% CI 0.70–1.03) nor PFS (HR 0.84, 95% CI 0.70–1.02) compared to the placebo group [72].

In the KEYNOTE-859 trial, the superiority of pembrolizumab plus chemotherapy (CAPOX or FP) over chemotherapy alone in patients with previously untreated HER2-negative mG/GEJ cancer was evaluated. Patients were eligible regardless of CPS status. Pembrolizumab plus chemotherapy significantly improved median OS vs. chemotherapy alone in the intention-to-treat (ITT) population (12.9 vs. 11.5 months; HR 0.78; 95% CI 0.70–0.87), CPS ≥ 10 group (15.7 vs. 11.8 months; HR 0.65; 95% CI 0.53–0.79), and CPS ≥ 1 group (13.0 vs. 11.4 months; HR 0.74; 95% CI 0.65–0.84). Similarly, median PFS was prolonged in the ITT population (6.9 vs. 5.6 months; HR 0.76; 95% CI 0.67–0.85), CPS ≥ 10 group (7.8 vs. 5.6 months; HR 0.62; 95% CI 0.51–0.76), and CPS ≥ 1 group (6.9 vs. 5.6 months; HR 0.72; 95% CI 0.63–0.82). Moreover, the ORR was significantly higher in the ITT population (51.3% vs. 42.0%), CPS ≥ 10 group (60.7% vs. 43.2%), and CPS ≥ 1 group (52.1% vs. 42.6%) [9].

Nivolumab plus fluoropyrimidine- and platinum-based chemotherapy is recommended for untreated HER2-negative mGC with CPS ≥ 5 and suggested for those with CPS 1–5 in the US and European countries [1,2,10]. Pembrolizumab plus fluoropyrimidine- and platinum-based chemotherapy is recommended for HER2-negative mGC with CPS ≥ 1 in the US and European countries [2,10]. In Japan, both nivolumab plus chemotherapy and pembrolizumab plus chemotherapy are recommended regardless of CPS, based on the consistent survival benefit observed across the overall randomized population in clinical trials [3].

The incidence of each treatment-related AE was comparable with or without ICI, and no new safety signals were identified in these trials [7,8,9,20]. Several immune-related adverse events (irAEs) have been reported, including gastrointestinal, hepatic, pulmonary, renal, and cutaneous events. Although 8–18% of patients treated with ICI plus chemotherapy experienced any grade ≥3 irAEs, most irAEs were grade 1–2 (Table 1). A total of 3% of patients treated with pembrolizumab plus chemotherapy discontinued treatment due to irAEs in the KEYNOTE-859 trial [9]. Regarding ICI plus chemotherapy, severe irAEs may interrupt not only the ICI dose but also the cytotoxic chemotherapy dose. Since the onset of irAEs varies from days to months after ICI initiation, even after ICI discontinuation [73,74,75,76,77], it is necessary to pay attention to irAEs.

In CheckMate-649 and KEYNOTE-859, the impact of ICI plus chemotherapy on health-related quality of life (HRQoL) was analyzed. ICI plus chemotherapy significantly improved HRQoL from baseline during treatment. Moreover, HRQoL in patients treated with ICI plus chemotherapy tended to be better than those treated with chemotherapy alone, particularly in the CPS ≥ 5 group [7,9,78,79].

## 4. Zolbetuximab Plus Chemotherapy for CLDN-Positive Gastric Cancer

Zolbetuximab is a first-in-class chimeric immunoglobulin G1 (IgG1) monoclonal antibody that specifically binds to an extracellular domain of CLDN 18.2, activating antitumor effects through antibody-dependent cellular cytotoxicity (ADCC) and complement-dependent cytotoxicity (CDC) [51,80,81,82].

The efficacy of zolbetuximab monotherapy in patients with previously treated HER2-negative CLDN-positive mG/GEJ cancer was evaluated in the phase IIa MONO trial. Those with CLDN 18.2 membrane staining with an IHC score of 2+ or 3+ in ≥50% of tumor cells were eligible for this trial. The ORR was 9% in the full analysis set population. In the patient subgroup with CLDN expression in ≥70% of tumor cells, a higher ORR (14%) was demonstrated [83].

The FAST trial was a randomized phase IIb trial evaluating the efficacy and safety of zolbetuximab plus EOX (epirubicin, oxaliplatin, and capecitabine) in patients with previously untreated HER2-negative mG/GEJ cancer. Those with CLDN 18.2 membrane staining with an IHC score of 2+ or 3+ in ≥40% of tumor cells were eligible for this trial. Patients were stratified according to the proportion of tumor cells expressing CLDN, at 40–69% or ≥70%. Zolbetuximab plus EOX significantly prolonged median PFS (7.5 vs. 5.3 months; HR 0.44; 95% CI 0.29–0.67) and median OS (13.0 vs. 8.3 months; HR 0.55; 95% CI 0.39–0.77) compared with EOX alone in the overall randomized population. In patients with CLDN expression in ≥70% of tumor cells, median PFS (9.0 vs. 5.7 months; HR 0.38; 95% CI 0.23–0.62) and median OS (16.5 vs. 8.9 months; HR 0.50; 95% CI 0.33–0.74) were improved with zolbetuximab plus EOX. In contrast, the difference in median PFS (4.3 vs. 4.1 months; HR 0.71; 95% CI 0.32–1.57) and median OS (8.3 vs. 7.4 months; HR 0.78; 95% CI 0.40–1.49) was not significant in patients with CLDN expression in 40–69% of tumor cells [58]. The current CLDN-positive criterion, defined as membrane staining with an IHC score of 2+ or 3+ in ≥75% of tumor cells, was established based on FAST trial results, although the cutoff point slightly differed due to assay variations between this trial and subsequent phase III trials [13,14,51].

SPOTLIGHT was a randomized phase III trial investigating the efficacy and safety of zolbetuximab plus FOLFOX in patients with previously untreated, HER2-negative, CLDN-positive mG/GEJ cancer. Zolbetuximab plus chemotherapy significantly prolonged median PFS (10.61 vs. 8.67 months; HR 0.75; 95% CI 0.60–0.94) and median OS (18.23 vs. 15.54 months; HR 0.75; 95% CI 0.60–0.94) compared with chemotherapy alone. The subgroup analysis for PFS and OS showed that zolbetuximab plus chemotherapy tended to have better outcomes than chemotherapy alone, except for patients with GEJ cancer. There was no significant difference in ORR with or without zolbetuximab (60.7% vs. 62.1%) [13].

GLOW was a phase III randomized trial that evaluated the efficacy and safety of zolbetuximab plus CAPOX in patients with previously untreated HER2-negative, CLDN-positive mG/GEJ cancer. Similar to the SPOTLIGHT trial, zolbetuximab plus chemotherapy significantly prolonged median PFS as the primary endpoint (8.21 vs. 6.80 months; HR 0.687; 95% CI 0.544–0.866) and median OS as the secondary endpoint (14.39 vs. 12.16 months; HR 0.771; 95% CI 0.615–0.965) compared to chemotherapy alone. As in the SPOTLIGHT trial, subgroup analysis showed that the survival benefit of zolbetuximab plus CAPOX tended to be worse than that of CAPOX alone in the GEJ group. There was no significant difference in ORR with or without zolbetuximab (53.8% vs. 48.8%) [14]. As these two trials consistently demonstrated the superiority of chemotherapy combined with zolbetuximab over chemotherapy alone in terms of OS and PFS, zolbetuximab plus chemotherapy was approved as a first-line treatment option for HER2-negative CLDN-positive mGC in Japan in 2024, followed by the US and European countries.

Regarding adverse events, nausea, vomiting, decreased appetite, and hypoalbuminemia were more frequently observed with zolbetuximab plus chemotherapy compared to chemotherapy alone. Any grade nausea and vomiting were observed in 76% and 67% of patients treated with zolbetuximab plus chemotherapy, and grade ≥3 nausea and vomiting were observed in 13% and 14% of the zolbetuximab plus chemotherapy group in the pooled analysis of SPOTLIGHT and GLOW [21]. However, 5% of patients treated with zolbetuximab plus chemotherapy in these trials discontinued zolbetuximab due to nausea or vomiting in the first 9 weeks [84].

Nausea and vomiting caused by zolbetuximab are hypothesized to be related to gastric mucosal damage resulting from ADCC and CDC antitumor activity targeting CLDN 18.2, which is also expressed in normal gastric epithelium [57,58,80,83] and gastric tissue damage after zolbetuximab injection was reported in ferrets [85]. The incidence of vomiting was relatively lower in patients with previous gastrectomy than in those without gastrectomy [13,83]. Since the mechanism of nausea and vomiting caused by zolbetuximab may differ from the mechanism of other antitumor agents, a more strategic approach is needed to manage them.

The pooled analysis of SPOTLIGHT and GLOW showed that the incidence of nausea/vomiting was lower in patients receiving 5-hydroxytryptamin receptor antagonists (5-HT3) + neurokinin-1 receptor antagonists (NK-1) + corticosteroids +/− other antiemetics compared to those receiving fewer prophylactic medications. Prophylactic corticosteroid as an antiemetic was administered to 44% of patients in SPOTLIGHT and 34% of patients in GLOW; however, corticosteroid use was not considered to affect the survival benefit in PFS and OS in patients treated with zolbetuximab plus chemotherapy [84]. Therefore, prophylactic antiemetics, including 5-HT3 + NK-1 + corticosteroids + other antiemetics (such as olanzapine), may also be beneficial in clinical practice.

Given that a higher infusion rate was associated with nausea and vomiting, stepwise infusion rates of zolbetuximab are recommended. Zolbetuximab is administered at the initial infusion rate during the first 30 to 60 min, and the infusion rate can be gradually increased if patients tolerate it. If nausea or vomiting occurs, infusion rate modification and the infusion interruption of zolbetuximab are required. As a result, a longer zolbetuximab infusion time is required in the early cycles, during which nausea and vomiting are frequently observed. Although the median total infusion time of zolbetuximab converges to 150 min in subsequent cycles, it takes 202 to 206 min in the early cycles [83,84]. A two-hour oxaliplatin infusion follows zolbetuximab infusion; therefore, the total infusion time for zolbetuximab plus chemotherapy may extend to several hours. The early cycles of zolbetuximab plus chemotherapy generally require inpatient administration in many cases.

The impact of zolbetuximab plus chemotherapy on HRQoL was analyzed based on results from SPOTLIGHT and GLOW. HRQoL was maintained during treatment, although no significant improvement was observed compared to chemotherapy alone [57].

## 5. Proposed Treatment Selection for HER2-Negative mGCs Expressing CLDN 18.2 and PD-L1

Biomarker analysis of tumor samples from patients screened for SPOTLIGHT or GLOW eligibility demonstrated that approximately 13–22% of CLDN-positive mGCs are also PD-L1 positive (CPS ≥ 5) [13,14,16]. Furthermore, several retrospective studies reported that approximately 26–74% were PD-L1 CPS ≥ 1, and approximately 5–17% were dMMR among CLDN-positive mGCs. The prevalence of these biomarkers was similar between CLDN-positive mGCs and CLDN-negative mGCs [13,14,15,16,17,18,19] (Table 2).

**Figure 1:**
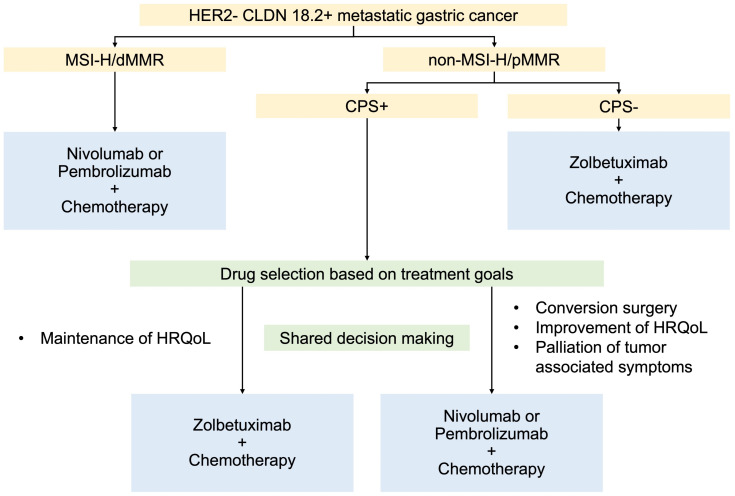
Proposed treatment algorism in patients with HER2-negative CLDN-positive metastatic gastric cancer. CLDN 18.2: claudin 18.2, CPS: combined positivity score, HER2: human epidermal growth factor receptor 2, HRQoL: health-related quality of life, MMR: mismatch repair, dMMR: deficient MMR, pMMR: proficient MMR, and MSI-H: microsatellite instability-high.

It has been suggested that CLDN-positive tumors are associated with an immunologically cold microenvironment and lower sensitivity to ICIs [18,51]; however, a recent retrospective study demonstrated that the survival benefit with nivolumab plus chemotherapy is not affected by CLDN expression [19]. Therefore, CLDN-positive mGC patients who have positive predictive biomarkers for ICI, CPS, or MSI-H/dMMR may benefit from ICI plus chemotherapy. Since there are no direct comparative studies between zolbetuximab and ICIs in combination with chemotherapy, selecting the optimal treatment for CLDN-positive patients with overlapping biomarker expression, CPS, and/or MSI-H/dMMR remains controversial (Figure 1). Although a direct comparison is not feasible due to different backgrounds, the results from each clinical trial may inform treatment selection. The outcomes of these trials are summarized in Table 3 [7,9,13,14,20,48].

### 5.1. For MSI-H/dMMR

Several previous trials have demonstrated remarkable benefits of ICI, with or without chemotherapy, for MSI-H/dMMR mGC patients. In the phase III KEYNOTE-062 trial, the efficacy of pembrolizumab monotherapy and pembrolizumab plus chemotherapy was evaluated vs. chemotherapy alone as first-line treatment for patients with CPS-positive mGC (CPS ≥ 1). Patients with MSI-H tumors tended to experience greater benefits from both pembrolizumab monotherapy and pembrolizumab plus chemotherapy compared to patients with non-MSI-H tumors [49,72]. KEYNOTE-158 was a phase II tumor-agnostic trial that evaluated the efficacy of pembrolizumab monotherapy in previously treated patients with MSI-H/dMMR tumors. In this trial, the ORR was 45.8%, and four cases showed complete response among patients with mGCs [50].

As summarized in Table 3, nivolumab or pembrolizumab plus chemotherapy showed more favorable HRs for OS (0.21–0.37) in the MSI-H subgroup compared with other subgroups in phase III trials, demonstrating that patients with MSI-H/dMMR may derive greater survival benefit from ICI plus chemotherapy [9,20,48,72]. A meta-analysis supports that patients with MSI-H tumors are more likely to benefit from ICI plus chemotherapy than those with non-MSI-H tumors [86]. Conversely, the association between MSI-H/dMMR and the efficacy of zolbetuximab plus chemotherapy remains uncertain due to limited data. Given that MSI-H/dMMR is a strong predictive factor, ICI plus chemotherapy is considered the preferred treatment option over zolbetuximab plus chemotherapy for patients with MSI-H/dMMR.

### 5.2. For Non-MSI-H/Proficient Mismatch Repair (pMMR)

The linear correlation between CPS and HRs for OS and PFS in patients treated with ICI plus chemotherapy has also been demonstrated in a meta-analysis of seven phase III randomized clinical trials [23]. HRs for OS and PFS in the CPS ≥ 10 group treated with ICI plus chemotherapy tended to be better than those in the ITT population treated with zolbetuximab plus chemotherapy (Table 3). ICI plus chemotherapy may provide better outcomes than zolbetuximab plus chemotherapy, particularly for patients with CPS ≥ 10. For patients with non-MSI-H/proficient mismatch repair (pMMR) tumors, treatment selection should be considered based on individual treatment goals, taking into account the efficacy profile differences of each therapy.

One of the differences in efficacy between zolbetuximab and ICI is observed in tumor response. Both nivolumab and pembrolizumab combined with chemotherapy improved ORR by approximately 10–15% compared to chemotherapy alone; however, no improvement in ORR was observed with zolbetuximab (Table 3). ICI plus chemotherapy would be the preferred treatment option when tumor response is required.

Although its clinical utility has not yet been established, the role of surgical intervention has been explored for GCs initially diagnosed as unresectable after curative resection becomes feasible due to chemotherapy. A retrospective study (CONVO-GC-1) demonstrated that R0 resection following chemotherapy improves survival time [87]. The survival benefit of surgical intervention following chemotherapy has been investigated, particularly in patients with limited metastasis, known as oligometastasis [88,89]. Evidence regarding surgical intervention following ICI plus chemotherapy is limited to several case reports [90,91,92]. However, ICI plus chemotherapy may contribute to achieving curative resection, especially for patients with oligometastasis, considering that tumor volume reduction and down-staging can be expected with ICI plus chemotherapy due to its benefit in tumor response.

Patients with mGC typically experience tumor-related symptom burden, including pain, ascites, abdominal distention, loss of appetite, gastrointestinal obstruction, and fatigue, which worsen with disease progression. First-line cytotoxic chemotherapy can maintain or improve patient-reported outcomes with HRQoL scores and reduce symptom burden [93]. Nivolumab plus chemotherapy improves HRQoL from baseline during treatment and significantly reduces the risk of HRQoL deterioration [7,78]. Zolbetuximab plus chemotherapy did not demonstrate superior HRQoL improvement compared to chemotherapy alone [57]. Therefore, ICI plus chemotherapy may be a superior treatment option for reducing tumor-related symptom burden. Considering that approximately 5% of patients treated with zolbetuximab plus chemotherapy discontinued zolbetuximab due to nausea and vomiting in the first three cycles [84], zolbetuximab may be unfavorable for patients who initially experience nausea or vomiting as a tumor-related symptom.

If tumor burden reduction is not required, zolbetuximab may be the preferred choice. CLDN positivity is a more reliable biomarker than CPS positivity. Moreover, the relatively lower risks of unexpected AEs may be an advantage of zolbetuximab plus chemotherapy compared to ICI plus chemotherapy. However, the initial cycles of zolbetuximab generally require infusion rate adjustment and infusion interruption to control nausea and vomiting [84]. This results in a prolonged injection time of several hours and the need for hospitalization, which may lead to time toxicity. The increase in time toxicity and the time spent in the hospital due to treatment may lead to a loss of time that patients can dedicate to their daily activities [94]. Therefore, ICI plus chemotherapy may be a viable treatment option for select patients. However, it is important to note that ICI plus chemotherapy may require discontinuation of the anticancer cytotoxic drug itself due to irAEs, and there is a risk of irAEs occurring even after ICI discontinuation.

As treatment options for HER2-negative mGC expand, treatment selection becomes burdensome for patients. Recently, the importance of shared decision-making (SDM) has been recognized as an approach to support patients’ autonomous decision-making in clinical practice to meet their needs. In the SDM process, clinicians provide evidence-based information about treatment options and associated benefits and harms, and patients share information about their values and preferences. Consensus formation is encouraged through mutual information sharing [95,96]. Although there is no well-defined treatment strategy for patients with HER2-negative mGC expressing CLDN and PD-L1 CPS, treatment options should be selected individually based on previous studies and clinical trials, considering therapeutic goals.

## 6. Future Directions

Currently, the efficacy and safety of chemotherapy combined with zolbetuximab plus ICI for HER2-negative mGCs expressing CLDN and PD-L1 are under investigation. The ILUSTRO trial is a phase II non-randomized study comprising five cohorts, and patients with mG/GEJ cancers with high (≥75% of tumor cells) or intermediate (≥50% and <75% of tumor cells) CLDN 18.2 expression are eligible (NCT03505320). The results of cohort 3A, in which three patients were treated with zolbetuximab plus pembrolizumab as third- or later-line therapy, have been reported. No tumor response was observed, and stable disease was observed in two patients [97]. In the ongoing cohort4A/4B, patients received combination chemotherapy with mFOLFOX6, zolbetuximab, and nivolumab. The results of this cohort are anticipated [13,14,51,61]. If the trial demonstrates positive outcomes, combination chemotherapy with zolbetuximab and nivolumab may become a standard treatment for HER2-negative mGCs expressing CLDN 18.2 and PD-L1 in the future. However, this trial is ongoing, and the publication of the results is pending. The selection of treatment for this population remains an important clinical issue.

Fibroblast growth factor receptor 2b (FGFR2b) is emerging as another target molecule of mGCs. FGFR2b overexpression is observed in approximately 16% of HER2-negative mGCs [98]. Bemarituzumab is a humanized IgG1 kappa monoclonal antibody targeting the extracellular domain of FGFR2b, which inhibits downstream signaling and downregulates FGFR2b [99]. Although tyrosine kinase inhibitors targeting the fibroblast growth factor receptor family are frequently associated with hyperphosphatemia, the risk of hyperphosphatemia is considered lower with bemarituzumab because it does not inhibit fibroblast growth factor 23 (FGF23) signaling, the ligand responsible for phosphate and vitamin D metabolism [100]. In the phase II FIGHT trial, the efficacy and safety of bemarituzumab plus mFOLFOX6 for patients with HER2-negative FGFR2b-positive mGCs were analyzed. In patients with FGFR2b-overexpressing tumors (an IHC score of 2+ or 3+ in ≥10% of tumor cells), bemarituzumab plus mFOLFOX6 significantly improved median PFS (14.0 vs. 7.3 months; HR 0.43; 95% CI 0.26–0.73) and median OS (24.7 vs. 11.1 months; HR 0.52; 95% CI 0.31–0.85) compared with mFOLFOX6 alone [101]. Several trials evaluating bemarituzumab efficacy are ongoing: the phase III FORTITUDE-101 trial (NCT05052801), phase Ib/III FORTITUDE-102 trial (NCT05111626), and phase Ib/II FORTITUDE-103 trial (NCT05322577) [102,103,104]. FORTITUDE-101 and FORTITUDE-103 evaluate bemarituzumab plus chemotherapy vs. placebo plus chemotherapy. FORTITUDE-102 and FORTITUDE-103 evaluate bemarituzumab and nivolumab plus chemotherapy vs. placebo and nivolumab plus mFOLFOX6. It has been reported that approximately 20% of CLDN-positive GCs are FGFR2b-positive [105]. Although the prevalence remains unclear, evidence suggests that a subset of mGCs with FGFR2b overexpression express PD-L1 CPS [106]. Since FGFR2b overexpression can overlap with CLDN 18.2 or PD-L1 CPS, treatment selection for patients with HER2-negative mGC expressing multiple overlapping biomarkers could be further complicated.

## 7. Conclusions

A significant improvement in survival time is expected with zolbetuximab and ICIs in HER2-negative mGC that is double-positive for CLDN 18.2 and PD-L1 CPS, although optimal drug selection cannot be uniformly determined due to the lack of direct comparative clinical trials. It is important to consider patients’ background factors, including tumor burden, the possibility of conversion surgery, and their preferences, in order to clarify treatment goals and select the most appropriate drug regimen for each individual case through SDM.

## Figures and Tables

**Table 1:** The incidence of potentially immune-related adverse events with ICI plus chemotherapy.

	CheckMate-649 [7]		ATTRACTION-4 [8]		KEYNOTE-859 [9]	
Treatment	Nivo + CT		Nivo + CT		Pembro + CT	
Chemotherapy regimen	CAPOX or FOLFOX		SOX or CAPOX		CAPOX or FP	
Incidence of AEs (%)	Any Grade	Grade ≥ 3	Any Grade	Grade ≥ 3	Any Grade	Grade ≥ 3
Any	NA	NA	NA	18	27	8
Gastrointestinal/Colitis	34	5	35.9	5.8	3	2
Skin related	28	4	37.3	3.9	2	2
Hepatic	27	4	23.1	4.2	1	0.4
Endocrine	14	0.8	11.4	2.2	NA	NA
Hypothyroidism	NA	NA	NA	NA	15	0.1
Hyperthyroidism	NA	NA	NA	NA	6	0
Thyroiditis	NA	NA	NA	NA	1	0
Adrenal insufficiency	NA	NA	NA	NA	1	0.5
Pulmonary/Pneumonitis	5	2	3.3	1.1	3	1
Renal/Nephritis	4	0.9	2.5	0.3	0.5	0.5

AE, adverse event; irAE, immune-related adverse event; CT, chemotherapy; Nivo, nivolumab; Pembro, pembrolizumab; CAPOX, capecitabine + oxaliplatin; FOLFOX, 5-fluorouracil + levofolinate + oxaliplatin; SOX, S-1 (tegafur, gimeracil, and oteracil potassium) + oxaliplatin; FP, 5-fluorouracil + cisplatin; NA, not available.

**Table 2:** Prevalence of biomarkers overlapping with CLDN 18.2 in advanced gastric cancer.

		Jia et al. [18]		Pellino et al. [17]		Kubota et al. [15]		Combined Analysis of SPOTLIGHT and GLOW [16]		Kim et al. [19]	
Patients, *n* (%)		CLDN+	CLDN-	CLDN+	CLDN-	CLDN+	CLDN-	CLDN+	CLDN-	CLDN+	CLDN-
		*n* = 42 (52.5)	*n* = 38 (47.5)	*n* = 117 (33.4)	*n* = 233 (66.6)	*n* = 98 (24.0)	*n* = 310 (76.0)	*n* = 1730 (38.4)	*n* = 2777 (61.6)	*n* = 96 (47.1)	*n* = 108 (52.9)
Sex	Female	7 (16.7)	12 (31.6)	37 (31.6)	95 (40.8)	32 (32.7)	95 (30.6)	637 (36.8)	853 (30.7)	45 (46.9)	38 (35.2)
	Male	35 (83.3)	26 (68.4)	80 (68.4)	138 (59.2)	66 (67.3)	215 (69.4)	1093 (63.2)	1924 (69.3)	51 (53.1)	70 (64.8)
HER2	positive	9 (21.4)	13 (34.2)	17 (14.5)	35 (15.0)	15 (15.3)	43 (13.9)	NA	NA	NA	NA
	negative	33 (78.6)	25 (65.8)	100 (85.5)	198 (85.0)	83 (84.7)	267 (86.1)	NA	NA	NA	NA
PD-L1	CPS < 1	9 (21.4)	8 (21.1)	87 (74.4)	165 (70.8)	24 (25.8)	68 (23.2)	NA	NA	NA	NA
	CPS ≥ 1	33 (78.6)	30 (78.9)	30 (25.6)	68 (29.2)	69 (74.2)	225 (76.8)	NA	NA	NA	NA
	CPS < 5	18 (42.9)	16 (42.1)	96 (82.1)	183 (78.5)	54 (58.1)	142 (48.5)	495 (82.6)	NA	59 (62.1)	53 (51.5)
	CPS ≥ 5	24 (57.1)	22 (57.9)	21 (17.9)	50 (21.5)	39 (41.9)	151 (51.5)	104 (17.4)	NA	36 (37.9)	50 (48.5)
	CPS < 10	23 (54.8)	21 (55.3)	NA	NA	NA	NA	NA	NA	NA	NA
	CPS ≥ 10	19 (45.2)	17 (44.7)	NA	NA	NA	NA	NA	NA	NA	NA
MMR status	pMMR	36 (85.7)	33 (86.8)	102 (87.2)	194 (83.3)	93 (94.9)	291 (93.9)	NA	NA	NA	NA
	dMMR	6 (14.3)	5 (13.2)	15 (12.8)	39 (16.7)	5 (5.1)	19 (6.1)	NA	NA	9 (10.0)	16 (16.7)
Lauren classification	Diffuse type	12 (28.6)	22 (57.9)	47 (40.2)	70 (30.0)	47 (48.0)	137 (44.2)	553 (33.3)	592 (24.2)	25 (26.0)	25 (23.1)
	Intestinal type	16 (38.1)	6 (15.8)	54 (46.2)	132 (56.7)	51 (52.0)	173 (55.8)	308 (18.6)	486 (19.9)	71 (74.0)	83 (76.9)
	Mixed /Other	14 (33.3)	10 (26.3)	14 (12.0)	25 (10.7)	NA	NA	386 (23.2)	603 (24.7)	NA	NA

CLDN, claudin; HER2, human epidermal growth factor receptor 2; PD-L1, programmed death-ligand 1; CPS, combined positivity score; mismatch repair; pMMR, proficient MMR; dMMR, deficient MMR; IHC, immunohistochemistry; NA, not available.

**Table 3:** The efficacy of combination chemotherapy for advanced gastric cancer.

		SPOTLIGHT [13]		GLOW [14]		CheckMate-649 [7]		ATTRACTION-4 [8]		KEYNOTE-859 [9]	
Treatment		ZOL + CT	CT	ZOL + CT	CT	Nivo + CT	CT	Nivo + CT	CT	Pembro + CT	CT
CT regimen		mFOLFOX6		CAPOX		CAPOX or FOLFOX		SOX or CAPOX		CAPOX or FP	
Patients, *n*		283	282	254	253	789	833	362	362	790	789
median observation time, months		22.1	20.9	17.7	18.4	47.4	47.3	26.6		31.0	
mOS, months	ITT polulation	18.23	15.54	14.39	12.16	13.7	11.6	17.45	17.15	12.9	11.5
HR for OS (95%CI)		0.75 (0.60–0.94)		0.771 (0.615–0.965)		0.79 (0.71–0.88)		0.90 (0.75–1.08)		0.78 (0.70–0.87)	
	CPS ≥ 10	NA	NA	NA	NA	15.0	10.9	NA	NA	15.7	11.8
		NA		NA		0.66 (0.57–0.77)		NA		0.65 (0.53–0.79)	
	CPS ≥ 5	NA	NA	NA	NA	14.4	11.1	NA	NA	NA	NA
		NA		NA		0.69 (0.60–0.79)		NA		NA	
	CPS ≥ 1	NA	NA	NA	NA	13.8	11.3	NA	NA	13.0	11.4
		NA		NA		0.75 (0.66–0.84)		NA		0.74 (0.65–0.84)	
mPFS, months	ITT polulation	10.61	8.67	8.21	6.80	7.7	6.9	10.94	8.41	6.9	5.6
HR for PFS (95%CI)		0.75 (0.60–0.94)		0.687 (0.544–0.866)		0.79 (0.71–0.89)		0.70 (0.57–0.86)		0.76 (0.67–0.85)	
	CPS ≥ 10	NA	NA	NA	NA	NA	NA	NA	NA	8.1	5.6
		NA		NA		NA		NA		0.62 (0.51–0.76)	
	CPS ≥ 5	NA	NA	NA	NA	8.3	6.1	NA	NA	NA	NA
		NA		NA		0.70 (0.60–0.81)		NA		NA	
	CPS ≥ 1	NA	NA	NA	NA	7.5	6.9	NA	NA	6.9	5.6
		NA		NA		0.77 (0.68–0.88)		NA		0.72 (0.63–0.82)	
ORR, %	ITT polulation	61	62	53.8	48.8	58	46	57	48	51	42
	CPS ≥ 10	NA	NA	NA	NA	59	45	NA	NA	61	43
	CPS ≥ 5	NA	NA	NA	NA	60	45	NA	NA	NA	NA
	CPS ≥ 1	NA	NA	NA	NA	60	46	NA	NA	52	43

CT, chemotherapy; ZOL, zolbetuximab; Nivo, nivolumab; Pembro, pembrolizumab; mFOLFOX6, FOLFOX, 5-fluorouracil + levofolinate + oxaliplatin; CAPOX, capecitabine + oxaliplatin; SOX, S-1 (tegafur, gimeracil, and oteracil potassium) + oxaliplatin; FP, 5-fluorouracil + cisplatin; mOS, median overall survival; mPFS, median progression-free survival; ORR, overall response rate; HR, hazard ratio; CI, confidence interval; ITT, intention-to-treat; CPS, combined positivity score; NA, not available.

## Data Availability

No new data were created or analyzed in this study. Data sharing is not applicable to this article.
