# Correction: Miyajima, Y.; Kawakami, T. Treatment Selection for Patients with HER2-Negative Metastatic Gastric Cancer Expressing Claudin 18.2 and PD-L1. *Cancers* 2025, *17*, 1120

**DOI:** 10.3390/cancers17203384

**Published:** 2025-10-21

**Authors:** Yusuke Miyajima, Takeshi Kawakami

**Affiliations:** Division of Gastrointestinal Oncology, Shizuoka Cancer Center, Shizuoka 411-8777, Japan; y38.miyajima@gmail.com

## Error in Figure

In the original publication [1], there was a mistake in “Proposed treatment algorism in patients with HER2-negative CLDN-positive metastatic gastric cancer (Figure 1)” as published. First, “CPS−” and “CPS+” have been corrected to “non-MSI-H/pMMR” and “MSI-H/dMMR”, respectively. Second, the label “With fewer tumor burden” has been corrected to “Maintenance of HRQoL”. Third, “Oligometastasis” has been corrected to “Conversion surgery”. Fourth, “Nivolumab Pembrolizumab” has been corrected to “Nivolumab or Pembrolizumab”. Fifth, “With tumor associated symptoms” has been corrected to “Palliation of tumor associated symptoms”. Sixth, “Drug selection according to tumor burden and treatment goal” has been revised to “Drug selection based on treatment goals”. Finally, “Shared decision making” has been newly added to emphasize the importance of patient involvement in treatment selection. The corrected “Proposed treatment algorism in patients with HER2-negative CLDN-positive metastatic gastric cancer (Figure 1)” appears below. With this correction, the email address of Dr. Yusuke Miyajima has been updated. The authors apologize for any inconvenience caused and state that the scientific conclusions are unaffected. This correction was approved by the Academic Editor. The original publication has also been updated.

## Figures and Tables

**Figure 1:**
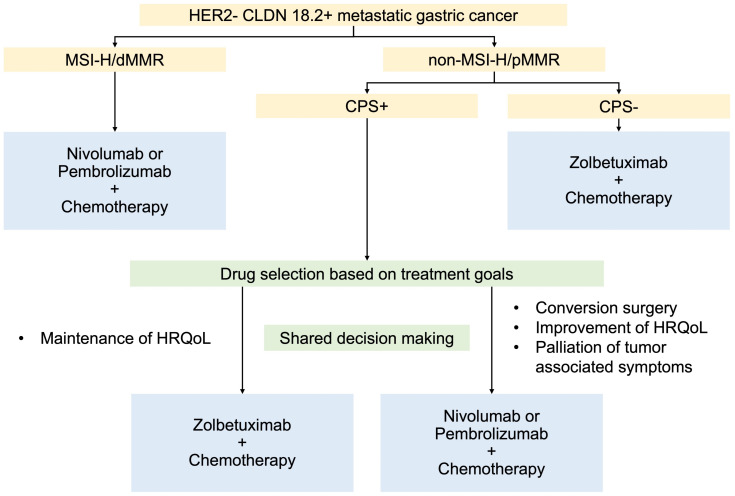
Proposed treatment algorism in patients with HER2-negative CLDN-positive metastatic gastric cancer. CLDN 18.2: claudin 18.2, CPS: combined positivity score, HER2: human epidermal growth factor receptor 2, HRQoL: health-related quality of life, MMR: mismatch repair, dMMR: deficient MMR, pMMR: proficient MMR, and MSI-H: microsatellite instability-high.
